# New strategy of personalized tissue regeneration: when autologous platelet concentrates encounter biomaterials

**DOI:** 10.3389/fbioe.2023.1297357

**Published:** 2023-11-22

**Authors:** Sicong Ren, Hanchi Wang, Sijia Ma, Jing Zhou, Jingjie Zhai, Yuemeng Zhu, Sheng Chen, Siyu Chen, Kewen Jia, Wenzhou Xu, Yanmin Zhou

**Affiliations:** ^1^ Department of Oral Implantology, Hospital of Stomatology, Jilin University, Changchun, Jilin, China; ^2^ Jilin Provincial Key Laboratory of Tooth Development and Bone Remodeling, Hospital of Stomatology, Jilin University, Changchun, Jilin, China; ^3^ Yiwu Stomatology Hospital, Yiwu, Zhejiang, China; ^4^ Department of Periodontology, Hospital of Stomatology, Jilin University, Changchun, Jilin, China

**Keywords:** biomaterials, PRP, APC, tissue, regeneration

## Abstract

Components in blood play an important role in wound healing and subsequent tissue regeneration processes. The fibrin matrix and various bioactive molecules work together to participate in this complex yet vital biological process. As a means of personalized medicine, autologous platelet concentrates have become an integral part of various tissue regeneration strategies. Here, we focus on how autologous platelet concentrates play a role in each stage of tissue healing, as well as how they work in conjunction with different types of biomaterials to participate in this process. In particular, we highlight the use of various biomaterials to protect, deliver and enhance these libraries of biomolecules, thereby overcoming the inherent disadvantages of autologous platelet concentrates and enabling them to function better in tissue regeneration.

## 1 Introduction

Tissue defects caused by tumors, trauma, inflammation and congenital diseases have long been a challenge in clinical treatment (Glyn-Jones et al., 2015; Ansarizadeh et al., 2019; Tang et al., 2020). Wound healing and tissue regeneration after defect formation is a dynamic and complex process (Ansarizadeh et al., 2019; Huang et al., 2022). Currently, the ideal approach is to use regenerative medicine strategies to promote wound healing and tissue regeneration. The key to regenerative medicine lies in the triad of tissue engineering: scaffold, cells and bioactive factors (Kim et al., 2020). Simply put, new tissue formation is facilitated by seeding cells and bioactive factors onto scaffold biomaterials (Zhao et al., 2022). There are some issues with this approach - traditional biomaterial scaffolds lack bioactivity, while the application of exogenous growth factor (GF) is costly (Faramarzi et al., 2018; Xie and Murphy, 2019), unsafe (Anitua et al., 2010; Faramarzi et al., 2018), and has unpredictable effects (Fernandes and Yang, 2016; Faramarzi et al., 2018). In light of this, autologous platelet concentrates (APC) have entered the view (Liu et al., 2017). APC is obtained by centrifuging blood. In order to take advantage of the therapeutic potential of biomolecules secreted by platelets, blood deconstruction can produce autologous platelets and plasma-derived fibrin scaffolds. Autologous GF, derived from platelets and plasma, interact with fibrin, extracellular matrix and tissue cells in a combined, synergistic and multidirectional manner to control the mechanism of tissue repair. As biological products derived from a patient’s own blood, they naturally contain key elements of tissue engineering: scaffold - the fibrin matrix formed by fibrinogen polymerization, whose unique inherent properties can directly facilitate wound healing by modulating cell behavior (Heher et al., 2018); bioactive factors - various GFs released by platelet degranulation during preparation, which synergistically promote tissue regeneration without side effects due to their endogenous source and optimal physiological ratios (Heher et al., 2018; Turer et al., 2020). However, APC is largely affected by the donor’s own conditions, such as age and poor vascular condition. There are some age-related changes in blood composition in the elderly, which is characterized by low concentrations of plasma and platelet-derived GF. In view of this situation, some scholars use allogeneic APC to treat elderly patients, showing only mild side effects while symptoms are improved, which partially opens the door for young blood donors to treat diseases as a source of allogeneic APC. In this way, allogeneic APC has become a safe and cost-effective treatment (Anitua et al., 2017).

Currently, various types of APC have been applied to different kinds of tissue defects, such as bone defects, periodontal tissue regeneration and soft tissue healing, and have demonstrated good therapeutic effects (Tang et al., 2020). However, systematic reviews contradict this efficacy, mainly due to the following disadvantages of APC: 1) fast degradation rate, inability to sustain long-term GF release (Zhang et al., 2021b; Ngah et al., 2021); 2) poor mechanical properties and mismatch with the complex regenerative environment (Allen et al., 2017; Isobe et al., 2017), especially when applied alone to bone defects (Ansarizadeh et al., 2019); 3) difficulty in maintaining long-term structural integrity (Ngah et al., 2021). To overcome these drawbacks, scaffold systems are needed to protect and stabilize APC, as well as modulate GF delivery, enabling better guidance of wound healing and tissue regeneration.

In biomimetic regenerative medicine, the combination of APC and biomaterial platform may be able to control the spatio-temporal and selective transmission of signal biomolecules. This strategy will enhance their therapeutic effectiveness and enhance tissue healing, transforming the normal repair response that usually leads to the formation of non-functional scar tissue into a regenerative result. Each specific combination of biomaterials and APC will show a unique biomolecule binding affinity and release curve, indicating that there are many engineering possibilities to explore in this field. In addition to the biological clues provided by APC, the inherent properties of biomaterials (physical, mechanical and chemical cues) can also increase the level of additional control over cell behavior, thereby synergistically enhancing the regeneration process (Mendes et al., 2018).

In this review, we provide a conceptual and investigative framework for modification methods of APC to enhance our understanding of these modification techniques and inform selection of different means to augment APC. First, we summarize and introduce the various types of APC and their characteristics. Next, we elaborate how APC function in each stage of wound healing and tissue regeneration, as well as the mechanisms involved. Finally, we detail biomaterial-based APC modification strategies and recent discoveries, providing direction for future advancements in this field.

## 2 Classification and characteristics of autologous platelet concentrates

**FIGURE 1:**
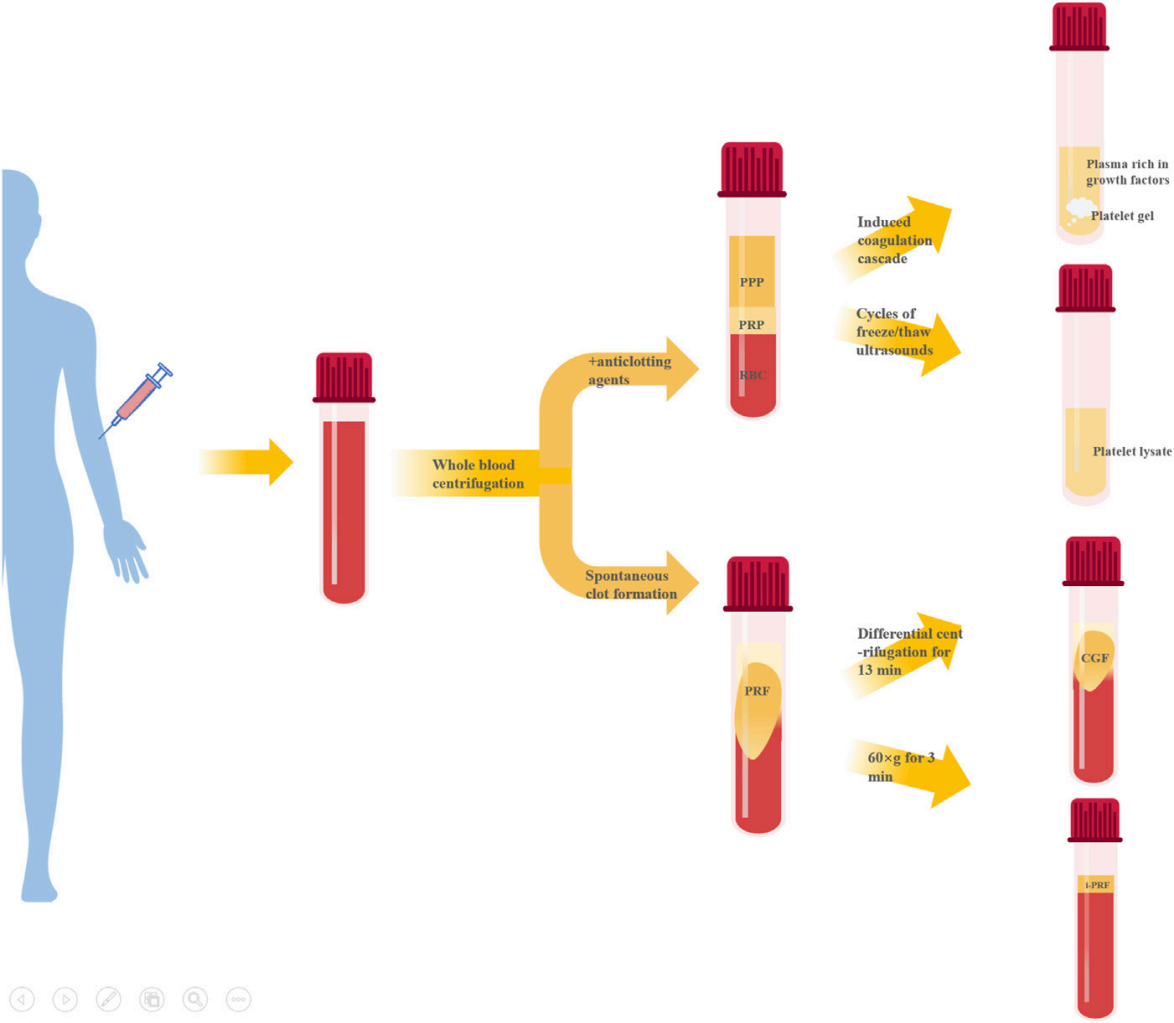
Classification of APC.

Preparation of APC starts with whole blood collection, typically via double centrifugation (Table 1). They can largely be divided into two categories based on whether anticoagulants are present during centrifugation - platelet-rich plasma (PRP) and platelet-rich fibrin (PRF). Further processing of PRP can produce platelet gel (PG), platelet lysate (PL), GF-rich plasma (PRGF), etc. Modulating centrifugation speed and time for PRF can generate concentrated growth factor (CGF), injectable PRF (iPRF), etc. (Figure 1).

**TABLE 1:** Preparation and character of APC.

Classification of APC		Preparation method	Addition of anticoagulants	Character
PRP and derivatives	PRP	Venous blood is collected in sterile tubes with anticoagulant, then centrifuged at low speed first to remove erythrocytes at the bottom. The upper plasma layer and buffy coat are transferred to new tubes and centrifuged at higher speed to obtain PRP after removing platelet-poor plasma	Yes	Liquid
	PG	Through the condensation induction of PRP	Yes	Gel
	PRGF	PRP is induced by condensation to collect the remaining liquid components	Yes	Liquid
	PL	Freeze-thaw cycle or ultrasound to break the platelets of PRP and collect liquid components	Yes	Liquid
PRF and derivatives	PRF	Put the blood sample without anticoagulant in the test tube and centrifuge immediately with 400 g for 10 min	No	Gel
	CGF	Venous blood was collected from each participant after providing informed consent; the tubes were immediately centrifuged in a special centrifuge device by 30 s acceleration, 2 min at 2,700 rpm (600 g), 4 min at 2,400 rpm (400 g), 4 min at 2,700 rpm (600 g), 3 min at 3,000 rpm, and 36 s deceleration	No	Gel
	iPRF	Put the blood sample without anticoagulant in the test tube and centrifuge immediately with 400 g for 10 min	No	Initially liquid and then spontaneously transformed into a gel state

### 2.1 Platelet-rich plasma and derivatives

#### 2.1.1 Platelet-rich plasma

PRP is typically prepared via double centrifugation or commercial kits. Venous blood is collected in sterile tubes with anticoagulant, then centrifuged at low speed first to remove erythrocytes at the bottom. The upper plasma layer and buffy coat (contain platelets and white blood cells) are transferred to new tubes and centrifuged at higher speed to obtain PRP after removing platelet-poor plasma (PPP) (Everts et al., 2020). Platelet activation in PRP induces α-granule degranulation, releasing various GFs like TGF-β, PDGF-AA, EGF, VEGF and FGF (Zhu et al., 2013). PRP is mainly composed of fibrin, platelets and white blood cells, its fibrin density is low, the connection is weak, the platelet collection efficiency is 40%–80%, the leukocyte collection efficiency is less than 40%, and the preservation of leukocytes and platelets is poor (Dohan Ehrenfest et al., 2009). However, their relative concentrations vary greatly among products, mainly due to: 1) donor variability; 2) pre-activation protocol; 3) platelet content. Consensus has not been reached on whether platelets require pre-activation before application nor which agent to use. Different commercial PRP systems may have varying separation methods, centrifugation speeds, collection systems and handling, leading to discrepancies in PRP composition and bioactivity (Arora and Arora, 2021).

Fibrin in PRP forms a tetramolecular bilateral connection, unfavorable for capturing GFs and cell migration. Previous studies show PRP fibrin networks are simple with premature fibers of smaller diameter (Dohan Ehrenfest et al., 2009), thus degrading faster than other APC with fewer GFs and leukocytes (Dohan Ehrenfest et al., 2009; Marin Fermin et al., 2023). However, the liquid state of PRP enables convenient combination with biomaterials.

#### 2.1.2 PG and PRGF

PG and PRGF are obtained by inducing PRP clotting (Pallotta et al., 2014; Anitua et al., 2015). The resultant gel clot is termed PG while the remaining liquid portion is PRGF (Pallotta et al., 2014). Clotting induction of PRP generates two products with distinct compositions and properties, providing more solutions when applied to different tissue defects. The composition of PRGF and PG is similar to that of PRP, but the use of calcium chloride instead of thrombin in the preparation process can release GFs more continuously, remove white blood cells and avoid the pro-inflammatory effects of proteases and acid hydrolases contained in white blood cells (Dohan Ehrenfest et al., 2009). Overall, PG has higher fibrin but lower GF content compared to PRGF. The clotting agent also affects the final product - collagenase produces gels with superior mechanics whereas thrombin yields products that release more GFs early on (Mendes et al., 2018).

#### 2.1.3 Platelet lysate

PL is a liquid biological solution obtained by disrupting platelets in PRP via freeze-thaw cycles or sonication (Oliveira et al., 2015; Qiu et al., 2021). The composition of PL is very complex, containing fibrin, plasma-derived proteins, GFs, cytokines, chemokines and mineral salts (Meftahpour et al., 2021). And the platelet membrane and white blood cells were removed. Advantages of PL include: 1) standardized preparation enabling higher GF concentration predictability; 2) no exogenous agents needed for PL and GF release (Tavelli et al., 2021); 3) ease of cold storage and transportation (Crespo-Diaz et al., 2011); 4) removal of erythrocyte debris and clots during preparation allows stable maintenance of liquid state for facile combination with biomaterials (Jafar et al., 2019; Qiu et al., 2021); However, PL has somewhat lower contents of certain GFs and fewer leukocytes compared to other APC, unfavorable for immune regulation (Allen et al., 2017).

### 2.2 Platelet-rich fibrin and derivatives

#### 2.2.1 Platelet-rich fibrin

Unlike PRP, PRF preparation requires no anticoagulants, bovine thrombin or other chemical additives, offering easier preparation and complete autologous sourcing. Blood without anticoagulant is centrifuged in tubes at 400 g for 10 min. The intermediate yellow gel obtained after centrifugation is PRF(Polak et al., 2019). Further compression and liquid expulsion can produce PRF membranes, providing multiple application formats. The composition of PRF is similar to that of PRP, but the fibrin density is higher, the connection is tighter, the collection efficiency of white blood cells and platelets is greater than 80%, and the two components are better preserved (Dohan Ehrenfest et al., 2009). Fibrin in PRF forms equilateral trimolecular connections, conferring a delicate, flexible fibrin network with high elasticity (Dohan Ehrenfest et al., 2006b), facilitating platelet and cytokine capture and cell migration (Dohan Ehrenfest et al., 2006a). PRF has thicker fibrin fibers, superior mechanics compared to PRP. PRF also has more leukocytes and GFs than PRP (Qiao et al., 2017), entrapped within the polymerizing fibrin network and slowly released during fibrin degradation. Additionally, PRF components display a gradient distribution with higher leukocyte, platelet, cytokine and GF concentrations towards the bottom that taper off upwards (Dohan Ehrenfest et al., 2006c).

#### 2.2.2 Concentrated growth factor

CGF, a next-generation APC, is prepared by altering centrifugation speed from PRP and PRF protocols (Qiao and An, 2017). Simply put, whole blood is separated into three layers by differential centrifugation and the intermediate yellow gel layer is CGF. CGF contains more GFs, fibrin and superior mechanics compared to PRP and PRF(Kabir et al., 2021; Zhang et al., 2023a). Abundant platelets and leukocytes also accumulate between CGF fibrin layer and erythrocyte layer (Kabir et al., 2021), exerting important immunomodulatory effects on tissue repair and healing (Chen and Jiang, 2020).

#### 2.2.3 Injectable platelet-rich fibrin

Recently, liquid iPRF has been developed using shorter centrifugation time and lower force (Pall et al., 2023). Venous blood without anticoagulant is centrifuged at 60 × g for 3 min and the pale-yellow supernatant is iPRF. Reduced time and speed decrease blood cell aggregation at the tube bottom, significantly increasing platelet and leukocyte content homogenously distributed within the dense fibrin network (Trzeciak et al., 2022). The low-speed centrifugation allows iPRF to remain injectable liquid initially for ∼15 min before gradually transforming into gel, enabling minimally invasive injection into defect sites or topical wound coating, as well as facile combination with biomaterials (Zhang et al., 2020).

In summary, compared to PRP products, PRF and its derivatives require no exogenous agents like anticoagulants during preparation, eliminating risks of infection, immune rejection and hypersensitivity while ensuring complete autologous sourcing and biocompatibility. GF, leukocyte and fibrin contents are also higher. Unlike the liquid state of PRP products, except for iPRF, PRF and CGF form gels after preparation, restricting combination modes with biomaterials. PRP products can interact with biomaterials more diversely, while freeze-dried powder or extract forms are more common for PRF and CGF biomaterial incorporation.

## 3 Roles of autologous platelet concentrates in different wound healing and tissue regeneration stages

**FIGURE 2:**
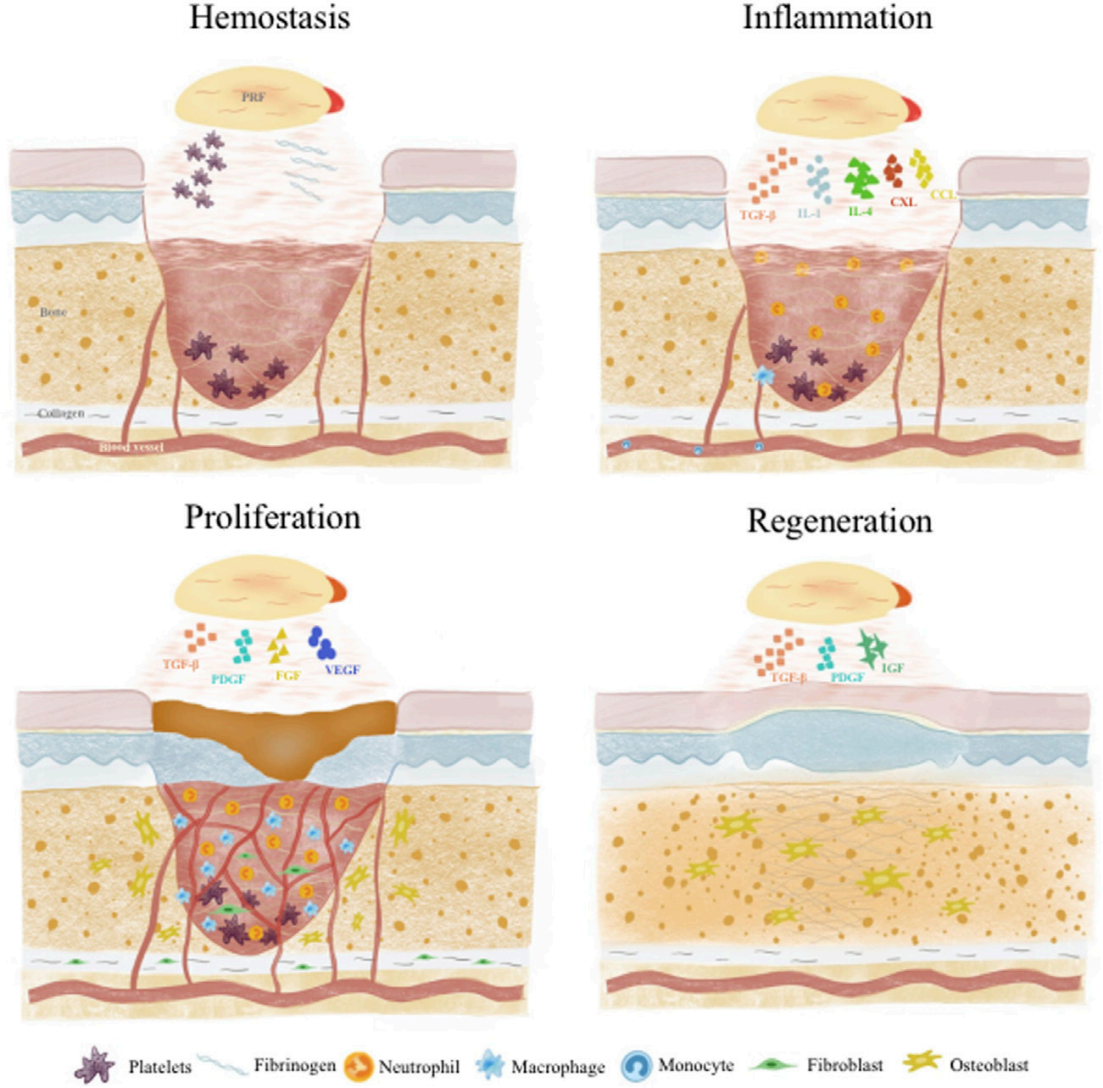
Roles of APC in different wound healing and tissue regeneration stages.

Wound healing and tissue regeneration follows a carefully orchestrated process that can be divided into 4 stages: 1) Hemostasis; 2) Inflammation; 3) Proliferation; 4) Remodeling. These phases overlap temporally in a complex interplay dynamically regulated by various cells and GFs (Xu et al., 2020; Kharaziha et al., 2021) (Figure 2).

### 3.1 Hemostasis

Injury induces immediate activation of coagulation cascades to prevent excessive blood and fluid loss. Platelets and fibrin in APC play vital roles in hemostasis. Platelets are central to primary hemostasis via adhesion, secretion and aggregation (Rodrigues et al., 2019). APC provide abundant platelets to participate in aggregation and facilitate primary hemostasis. APC factors also strongly support coagulation -vWF enables thrombin formation and inhibits fibrinolysis to ensure fibrin deposition and stability. GFs like platelet derived growth factor (PDGF) promote endothelial and mural cell proliferation to restore vascular integrity (Tang et al., 2023). Meanwhile, APC fibrinogen and fibrin reinforce fibrin networks and delay dissolution for more reliable hemostasis (Shiu et al., 2014). Fibrin can interact with platelet integrin α_IIb_β_3_ to form more stable blood clots (Heher et al., 2018). Besides these two main components, other APC constituents like albumin, globulin, fibronectin, clotting factors, P-selectin and electrolytes facilitate platelet-endothelial cell interplay, accelerate platelet aggregation and thrombus formation to achieve faster, sustained hemostasis (Amable et al., 2013; Ellis et al., 2018).

### 3.2 Inflammation

Following platelet plug and fibrin clot formation to effect hemostasis, inflammation ensues as neutrophils and monocytes are recruited to the wound site and differentiate into macrophages (Cooke, 2019). This inflammatory response helps remove foreign bodies, bacteria and damaged endogenous tissue, as well as secrete chemokines and GFs to attract more cells, guiding the transition into the next regenerative phase (Cooke, 2019; Raziyeva et al., 2021). Monocytes in APC bind fibrin through integrin Mac-1 to promote phagocytosis/debridement and regulate the development of M1 and M2 macrophages (Heher et al., 2018). APC GFs [e.g., transforming growth factor-β (TGF-β)] and cytokines [e.g., interleukin (IL)-1] support early inflammation by inducing monocyte recruitment and differentiation into macrophages for debris clearance and tissue repair (Scopelliti et al., 2022). Native APC neutrophils and monocytes also actively participate (Katz, 2021). Afterwards, APC cytokines like IL-4 polarize proinflammatory M1 macrophages into anti-inflammatory, pro-repair M2 macrophages, preventing excessive sustained inflammation and delayed healing (Uchiyama et al., 2021; Jayaram et al., 2023). Insulin growth factor-1 (IGF-1) can solve excessive inflammation by down-regulating NF-kB pathway and inhibiting the activity of macrophage migration inhibitory factor (MIF) (both related to M1 macrophage polarization), thus achieving a balance between inflammation and tissue regeneration (Anitua et al., 2019). APC components like tumor necrosis factor α (TNFα), C-X-C motif chemokine ligand (CXCL)4, CXCL7, CC motif chemokine ligand (CCL)3 and CCL5 also exert antimicrobial and immunomodulatory effects (Mussano et al., 2016; Everts et al., 2020).

### 3.3 Proliferation

This phase commences as fibroblasts migrate to form granulation tissue, providing support for neovascularization and subsequent tissue remodeling and regeneration. A key aspect of proliferation and the later tissue regeneration stage is sufficient vascularization. Cell proliferation and extracellular matrix formation relies on intricate interactions between newly formed vessels and surrounding cells, with adequate vascularization supplying oxygen and nutrients (Liang et al., 2021). APC TGF-β promotes fibroblast migration to facilitate granulation tissue (Paez-Mayorga et al., 2020) and dedifferentiate the epidermal cells through B1-Integrin/ILK pathway, so as to accelerate the re-epithelialization of skin wound healing (Anitua et al., 2019). IL-6 activates Janus kinase pathway, indirectly induces neutrophil and monocyte infiltration, fibroblast migration and collagen deposition, and promotes angiogenesis by inducing the production of TGF- β, IL-1, vascular endothelial growth factor (VEGF) and hepatocyte growth factor (HGF). IL-6 activates Janus kinase pathway, indirectly induces neutrophil and monocyte infiltration, fibroblast migration and collagen deposition, and promotes angiogenesis by inducing the production of TGF- β 1, IL-1, VEGF and HGF (Mendes et al., 2018). Abundant APC GFs like VEGF and fibroblast growth factor (FGF) enable endothelial cell migration into the granulation tissue to form new vessels (Etulain, 2018). Meanwhile, APC PDGF acts on mural cells to mature the nascent vasculature (Zamani et al., 2019). Most importantly, simultaneous presence of these APC GFs at optimal endogenous ratios enables synergistic effects on endothelial-mural cell interplay for enhanced vessel stability (Anitua et al., 2019). During remodeling, collagen production and degradation gradually equalizes and transitions from type III to type I collagen while cell populations like macrophages and fibroblasts taper off (Liang et al., 2021). APC cytokines, GFs and proteases all participate to generate a more suitable extracellular matrix (Mendes et al., 2018).

### 3.4 Tissue regeneration

Various APC GFs play important roles in tissue regeneration. PDGF potently chemoattracts mesenchymal stem cells, osteoblasts, fibroblasts and endothelial cells. It is the most potent osteogenic chemotactic GF, stimulating osteoblast DNA synthesis, proliferation, collagen and non-collagen protein production (Gao et al., 2021). PDGF also promotes endothelial proliferation and VEGF mRNA expression, reconstructing blood supply at injury sites (Cecerska-Heryc et al., 2022). TGF-β not only regulates osteoblast proliferation and differentiation by modulating cell division and proliferation, but also activates associated signaling pathways to induce mesenchymal stem cell osteogenic differentiation and osteoblast proliferation. It further stimulates osteoblast synthesis of extracellular matrix components including collagens, osteonectin and osteopontin, promoting matrix mineralization (Manokawinchoke et al., 2019). In addition, PDGF and TGF β in ABC also have a synergistic effect on osteogenic differentiation. PDGF positively regulates human mesenchymal stem cells (hMSC) osteogenic differentiation induced by TGF- β through the synergistic crosstalk between MEK and PI3K/Akt-mediated signal transduction (Yokota et al., 2014). IGF facilitates stem cell expression of alkaline phosphatase, RUNX2 and OPN while also participating in osteogenic differentiation (Wang et al., 2023a).

## 4 APC modification strategies based on biomaterials

Previously, APC application was largely limited to: 1) direct defect/wound application; 2) *in situ* gel formation via addition of thrombin or calcium chloride (Santos et al., 2022; Wang et al., 2023b). Disadvantages like uncontrolled GF release, fast fibrin degradation leading to structural instability, and difficulties with transportation and storage warrant introduction of scaffold biomaterials (hydrogels, nanofibers, microspheres) or biomaterial platforms (3D printing, semi-interpenetrating network, layer-by-layer self-assembly) to maintain structural stability and enable spatiotemporal, selective GF delivery. Interactions between these biomaterials and APC range from simple encapsulation to non-covalent binding (hydrogen bond, van der Waals forces, electrostatic interactions) and covalent conjugation (chemical crosslinking). These explorations not only provide the inherent biochemical cues of APC, but also cell behavior-modulating biophysical signals from the materials themselves, greatly promoting wound healing and tissue regeneration. In this section, we summarize these interesting modalities.

### 4.1 Hydrogel-based APC modification strategies

Hydrogels have attracted considerable attention as drug delivery scaffolds due to their high biocompatibility and tunable stiffness. The hydrated 3D polymer networks closely mimic native extracellular matrices, making hydrogels ideal for housing cells and bioactive agents to enhance tissue regeneration (Wang et al., 2020; Correa et al., 2021). Therefore, hydrogels are excellent scaffolds for controlled spatiotemporal, selective APC GF delivery.

**FIGURE 3:**
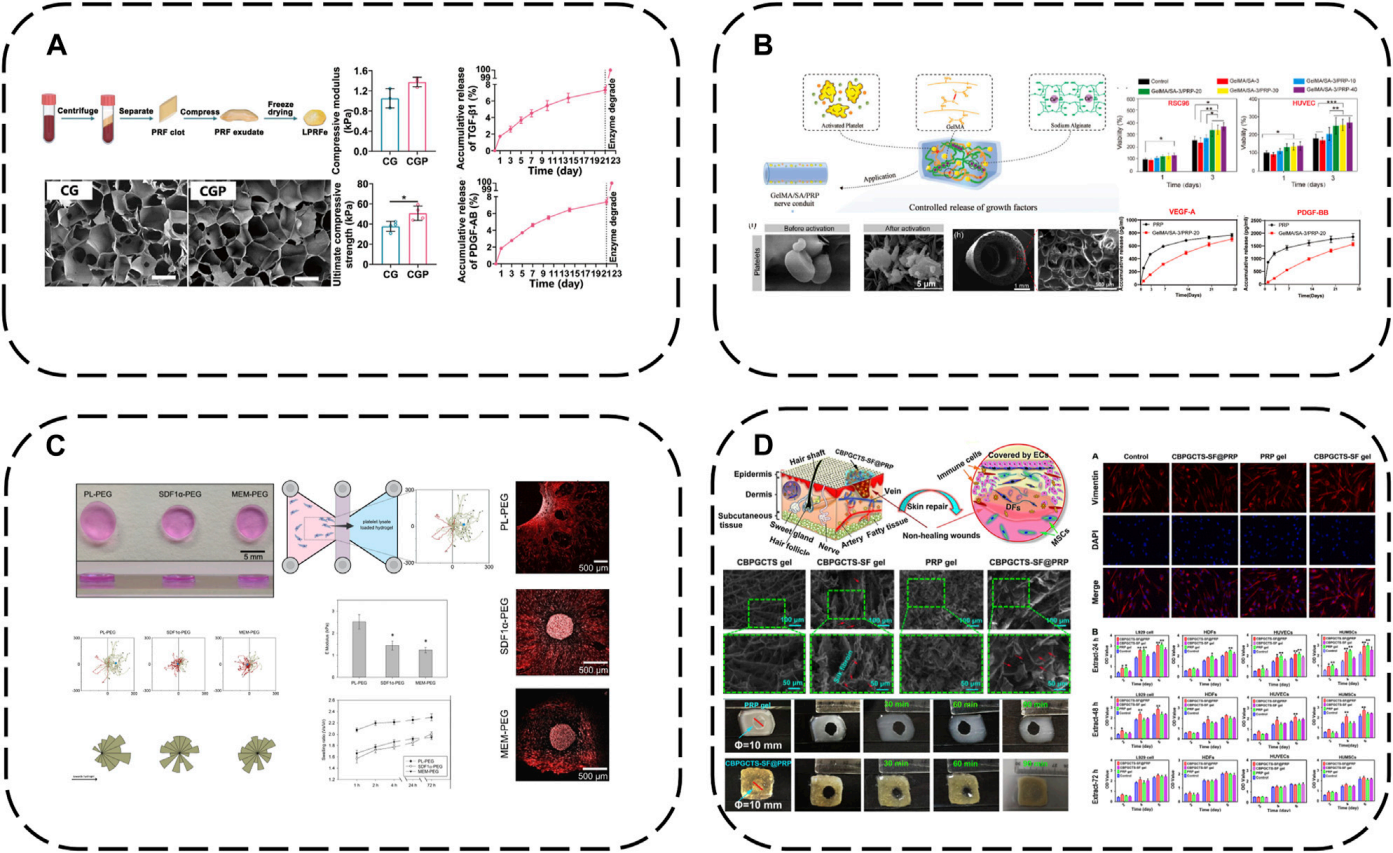
Hydrogel-based APC modification strategies: **(A)** Loaded LPRFe into CMCSMA/GelMA hydrogels; **(B)** Combining the GelMA/SA hydrogel with PRP forms GelMA/SA/PRP hydrogels; **(C)** PEG hydrogels act as reservoirs for PL; **(D)** Load PRP into CBPGCTS hydrogels.

In therapy, APC GFs typically display an initial burst release, thus sustaining release over prolonged tissue regeneration is key for efficacy. A straightforward approach is hydrogel encapsulation to retard APC degradation and enable controlled GF release. A recent representative study loaded lyophilized PRF exudate (LPRFe) into photocrosslinked carboxymethyl chitosan methacrylate (CMCSMA)/gelatin methacrylate (GelMA) hydrogels (Gan et al., 2023) (Figure 3A). Results showed LPRFe incorporation did not significantly affect CMCSMA/GelMA hydrogel morphology, swelling, mechanics or degradation. Cell compatibility was enhanced and APC addition promoted bone marrow mesenchymal stem cells (BMSCs) proliferation, adhesion, migration and osteogenic differentiation. Notably, the composite hydrogel enabled sustained APC GF release for up to 23 days and successfully repaired critical-sized rat bone defects, markedly accelerating bone repair.

To further enhance GelMA hydrogel mechanics, sodium alginate (SA) addition increases mechanical strength and degradation time. Combining the GelMA/SA hydrogel with PRP forms GelMA/SA/PRP hydrogels with excellent mechanics, degradation and bioactivity, sustaining release of two major PRP GFs, VEGF-A and PDGF-BB, for continuous microenvironmental modulation (Dong et al., 2023) (Figure 3B).

Similarly, poly (ethylene glycol) (PEG) hydrogels can act as reservoirs for GF-rich human PL, forming human PL-loaded PEG hydrogels (Chahal et al., 2021) (Figure 3C). This study showed PL-loaded PEG hydrogels enabled sustained GF release. PL conferred bioactivity, with scaffold-released biomolecules inducing and increasing hMSC directional migration.

Additionally, 4-formylbenzoic acid (CB) can functionalize PEG for reversible Schiff base crosslinking of carboxymethyl chitosan (GCTS) into dynamic hydrogels (crosslinked GCTS labeled CBPGCTS), then freeze-dried silk fibroin (SF) is incorporated by mixing to load PRP into CBPGCTS hydrogels, creating injectable, self-healing, physically stable CBPGCTS-SF@PRP composite hydrogels with anti-enzymatic degradation, sustainable PRP release and enhanced mesenchymal stem cell chemotaxis (Qian et al., 2020) (Figure 3D).

**FIGURE 4:**
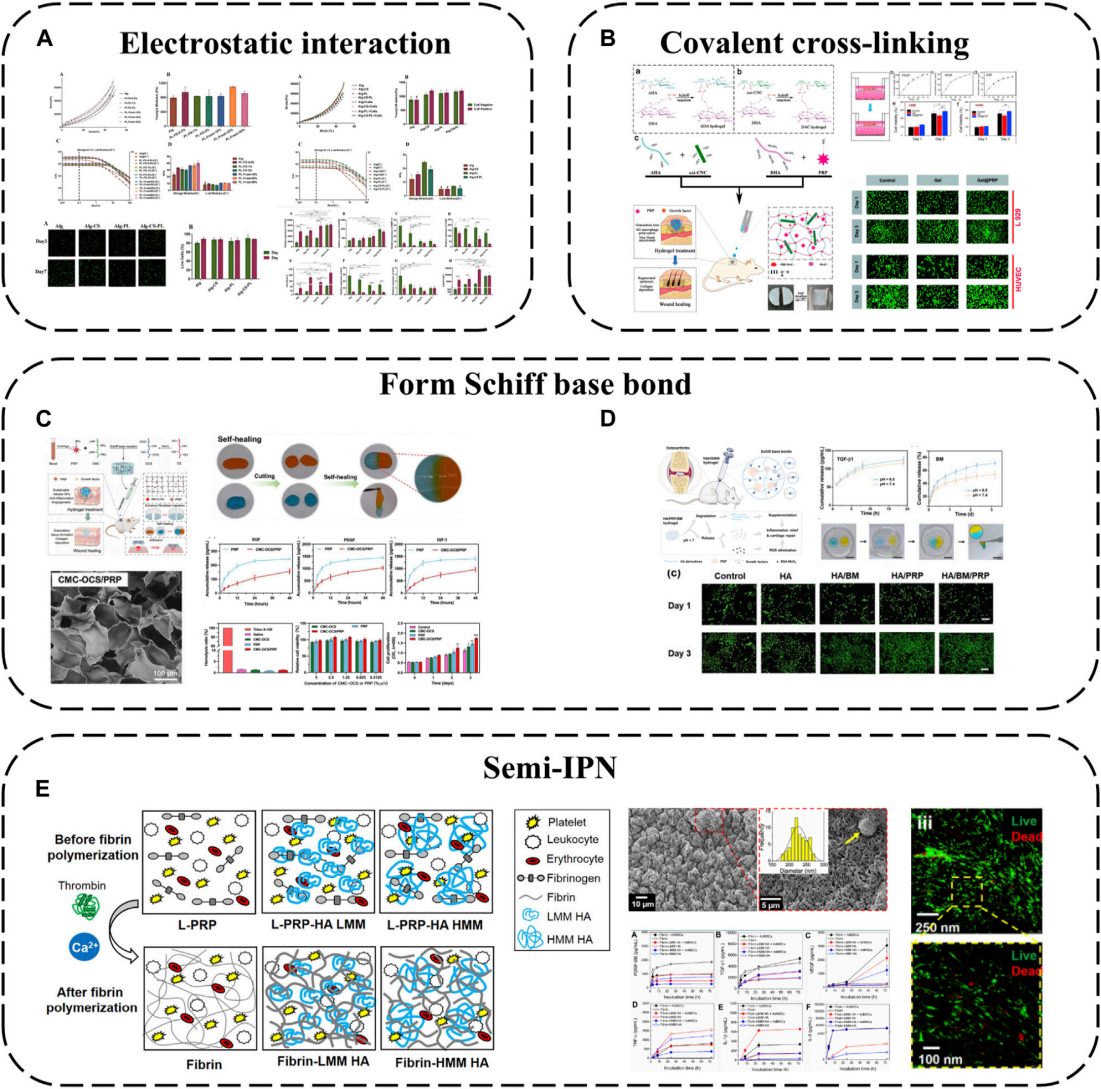
Hydrogel-based APC modification strategies: **(A)** Alginate-chondroitin sulfate-platelet lysate hydrogel; **(B)** PRP loaded into ADAC hydrogel; **(C)** Hydrogel formed by crosslinking PRP with OCS and CMC by Schiff base; **(D)** PRP is crosslinked BSA-MnO_2_ (BM) NPs by Schiff base to obtain HA/PRP/BM mixed hydrogel; **(E)** PRP-HA semi-IPNs prepared by controlled mixing of L-PRP with HA.

In addition to simply using hydrogel for packaging, some components of hydrogel can also be used to produce electrostatic interaction with APC, which can not only better absorb GFs, but also make APC have a gain effect on the structure of hydrogel. For example, alginate-chondroitin sulfate-PL hydrogel was prepared by mixing chondroitin sulfate (CS) and PL with sodium alginate (Alg) (Jahangir et al., 2020) (Figure 4A). The combination of PL and CS with Alg hydrogel could enhance the mechanical properties and Sertoli cell viability. The addition of PL significantly increased the proliferation of MSC and promoted the cartilage differentiation of MSC. The interaction between CS and Alg increases the negative charge, while the combination of clotting protein and positive charge of PL can enhance the cross-linking degree of the hydrogel, thus forming a stronger network, increasing its Young’s modulus and viscoelastic modulus, and better simulating the physiological environment of chondrocytes.

Using covalent bonds to anchor APC to the hydrogel network is a more effective strategy, which can better complete the spatial-temporal delivery of GF and greatly enhance the mechanical properties of APC. A kind of hyaluronic acid nanocomposite hydrogel based on aldehyde modified sodium hyaluronate (AHA), hydrazide modified sodium hyaluronate (ADA) and aldehyde modified cellulose nanocrystals (oxi-CNC) was developed (Li et al., 2022b) (Figure 4B). The composite hydrogel is developed through the reversible acyl Hydrazone bond formed between AHA, ADA and oxi-CNC. It not only has good mechanical properties but also has the ability of self-repair, so it can protect the biological activity of PRP through the rapid self-repair ability after damage. PRP can be loaded into ADAC through imine bonds formed between its amino groups and aldehyde groups on AHA or oxi-CNC to promote skin wound healing. Compared with individual hydrogels ADAC hydrogels can provide significant protection for PRP and provide controlled release of GFs.

Because APC contains a large number of amino groups, it is also a simple and effective strategy to use its amino groups to produce Schiff base reaction to combine with hydrogels. For example, extracellular matrix (ECM) biomimetic hydrogel formed by crosslinking PRP with oxidized chondroitin sulfate CS (OCS) and carboxymethyl chitosan (CMC) by Schiff base (Zhang et al., 2023b). Or PRP is crosslinked with hyaluronic acid (HA) containing BSA-MnO_2_ nano particles (BM-NPs) by Schiff base to obtain HA/PRP/BM mixed hydrogel (Zhou et al., 2022) (Figures 4C, D). The amino group in PRP reacts with the group in hydrogel by primary amine condensation to form dynamic Schiff base bond. Because of the self-repairing property and pH response ability of Schiff base bond, this kind of hydrogel not only has viscous supplement and self-healing ability, but also when exposed to weakly acidic inflammatory microenvironment, the encapsulated GF can be released from the hydrogel network to achieve on-demand wound healing. Rich active groups (single bond NH_2_, single bond OH, etc.) and controlled viscoelasticity make these hydrogels have high adhesion to the wound surface and high adaptability to irregular wounds.

Besides covalent or non-covalent binding with hydrogel components, semi-interpenetrating networks are another interesting combination approach. Semi-interpenetrating polymer networks (semi-IPN) comprise crosslinked and linear polymer forms. PRP-HA semi-IPNs can be prepared by controlled mixing of L-PRP with HA (de Melo et al., 2020) (Figure 4E). PRP-HA semi-IPNs comprise polymerized fibrin and HA coils wound among the fibers, physically crosslinked but not covalently bound to specific fibrin sites, with variable stacking and viscoelasticity. The fibrosis process of PRP-HA can be further controlled by adjusting the molecular weight of HA, which is convenient for clinical injection. Due to the increase of hydrophilic waters in the semi-interpenetrating network structure, the swelling rate of fibrin-HA is significantly higher than that of fibrin, which is beneficial to the diffusion of nutrients. The results of degradation experiments show that the mass loss of the composite hydrogel is less than 20% in the first 2 weeks, indicating that the semi-interpenetrating network has moderate structural stability. Although semi-interpenetrating networks form softer hydrogels than fibrin, they capture more white blood cells and GFs, allowing MSC to differentiate preferably in these microenvironments.

### 4.2 APC modification using microspheres and micro/nanoparticles

Microsphere technology attractively delivers cells/bioactive molecules by providing a protective shell and controlled release of GFs/drugs. Injected into tissues minimally invasively or distributed throughout engineered tissue matrices as extenders, the porous microsphere structures mimic native hierarchical organization, promoting cell migration and rapid vascularization. The structures also provide physical cues guiding cell proliferation and differentiation, while loaded biomolecules provide chemical signals (Sun et al., 2022).

Simple applications encapsulate or adsorb APC onto microspheres, e.g., using Alg microspheres as PRP delivery systems, co-loaded with adipose-derived stem cells (ADSCs) (Sun et al., 2022). PRP addition promotes encapsulated ADSC migration and osteogenic differentiation while also attracting cells from surrounding tissues to microsphere surfaces for enhanced vascularization and regeneration. Encapsulating PRP in Alg beads prevents enzymatic GF degradation and optimizes release in a controlled manner.

**FIGURE 5:**
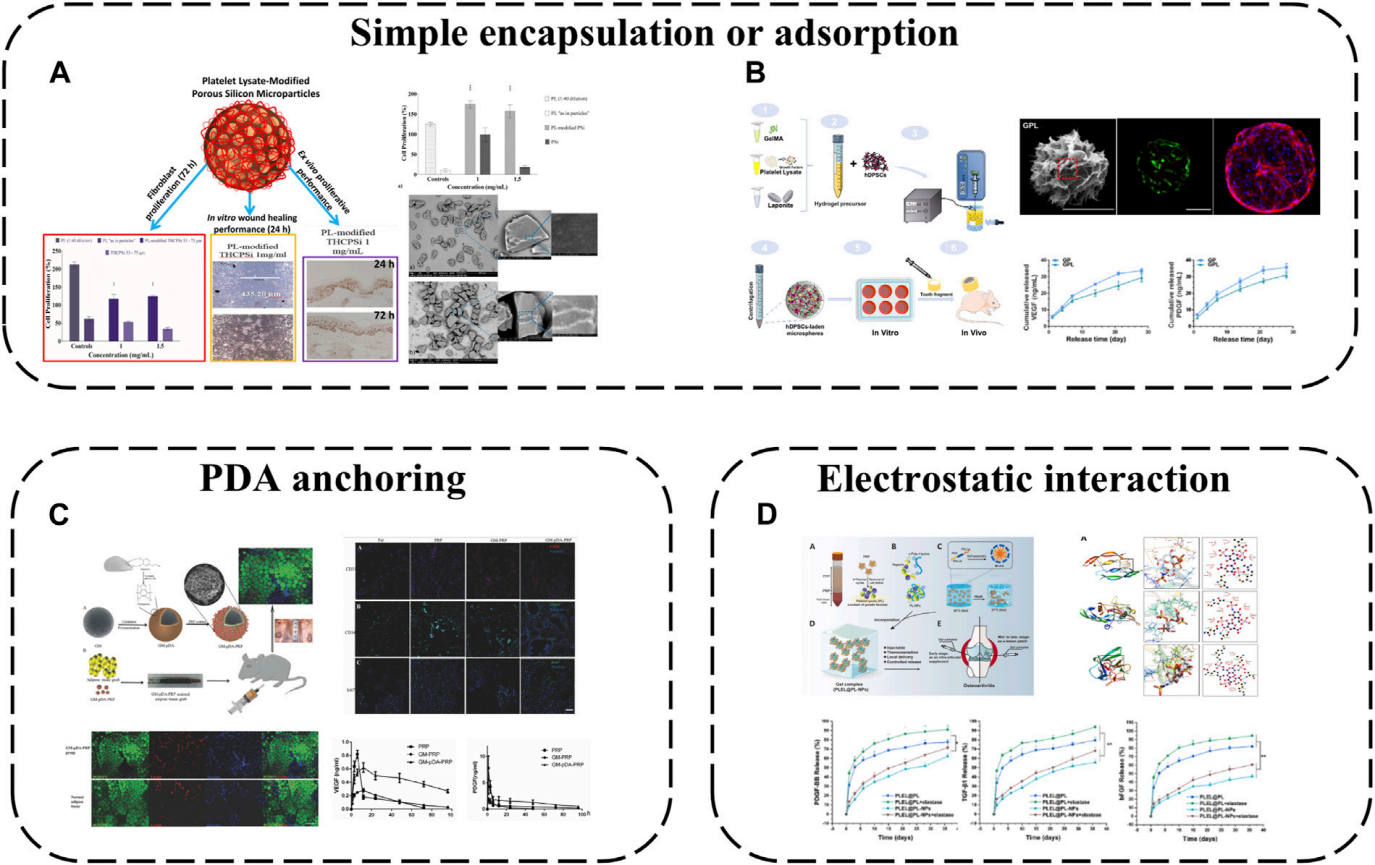
APC modification using microspheres and micro/nanoparticles: **(A)** A drug delivery system is obtained by loading PL onto PSi particles; **(B)** PL incorporated into GelMA microsphere; **(C)** PDA-mediated PRP immobilization onto gelatin microspheres; **(D)** Hep/EPL NPs combined with PL via electrostatic.

Mesoporous microns and nanoparticles as local drug delivery systems have been widely studied in different fields. Porous silicon (PSi) particles are excellent materials for controlled drug delivery applications because of their large pore size and easy functionalization of their surfaces with biomolecules. Therefore, a drug delivery system is obtained by loading PL onto PSi particles (Fontana et al., 2016) (Figure 5A). The results showed that PL modified PSi particles had cytocompatibility and enhanced cell proliferation in different experimental environments. This modification method improves the stability and applicability of PL to skin wounds and realizes continuous tissue regeneration.

PL was incorporated into GelMA microsphere system (GP) (Zhang et al., 2021a) (Figure 5B). Nano-clay Laponite was added to improve drug delivery capacity. Finally, GPL was prepared by electrostatic droplet technology. These hybrid microspheres have a suitable size range (180–380 μm). The Young’s modulus is about 3 -fold higher than that of traditional APC, and the swelling rate and degradation rate decrease at the same time. GF derived from PL was continuously released from GPL microspheres for 28 days. More importantly the released GF can effectively induce the tubule formation of human umbilical vein endothelial cells (HUVEC) and significantly improve the proliferation diffusion and odontogenic differentiation of encapsulated human dental pulp stem cells (hDPSC). The results of subcutaneous implantation further confirmed that GPL group enhanced microangiogenesis and pulp-like tissue regeneration. This work shows that the GelMA microsphere system mixed with PL is a promising functional carrier for promoting vascularized dental pulp regeneration.

While physical adsorption or encapsulation are safe delivery modalities without complex chemistry or bioactivity alterations, effective immobilization is not guaranteed. Chemical conjugation may alter biomolecule bioactivity, introduce toxic chemicals or require multiple reaction steps. Polydopamine (PDA)-mediated PRP immobilization onto gelatin microspheres (GM) is therefore a viable strategy to improve tissue regeneration (Zhou et al., 2017) (Figure 5C). GM sequesters PRP at the micro-scale while the highly adhesive PDA coating fixes more platelets to GMs, releasing more GFs for local delivery. The composite microspheres provide physical and chemical cues as biomimetic micro-niches for tissue regeneration while remaining injectable for convenient clinical use.

Polymeric micro/nanoparticles, especially heparin (Hep)/acetylated heparin-based NPs, are widely employed as protein drug carriers owing to their affinity for most GFs and excellent biocompatibility/degradability. Hep and ε-poly-L-lysine (EPL) NPs have thus been combined with PL via electrostatic self-assembly into PL-NPs (Tang et al., 2021) (Figure 5D). Hep/EPL NPs improve PL release behavior and provide controlled release with sustained GF delivery. Direct APC introduction into gels may disrupt sol-gel transitions by aggregating with oppositely charged species and precipitating, but this modification enhances APC dispersibility, maintaining initial gelation capacity and improving stability when incorporated into hydrogel systems.

### 4.3 APC modification using electrospinning

**FIGURE 6:**
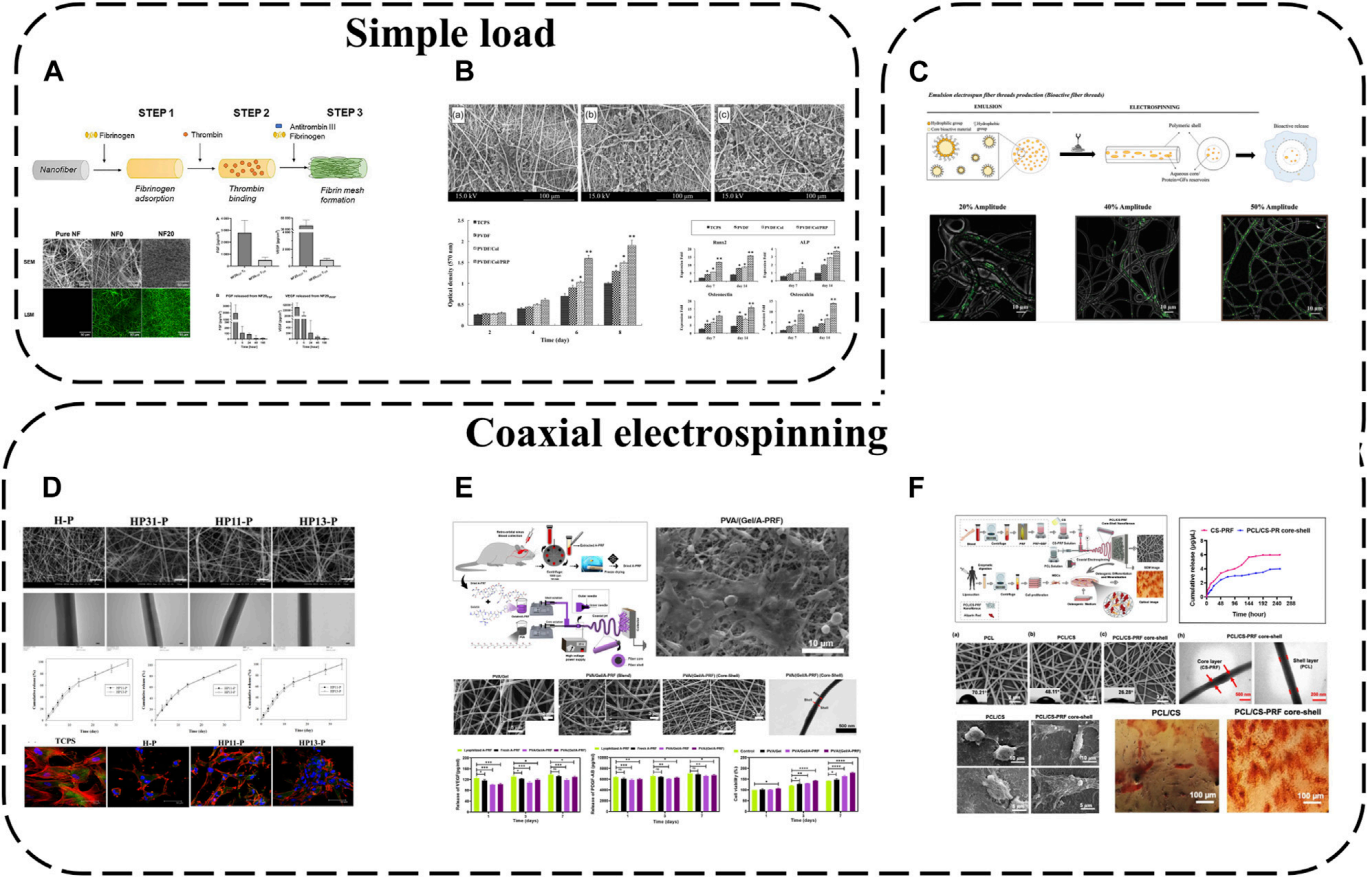
APC modification using electrospinning: **(A)** PL used to functionalize PLCL/PCL nanofibers; **(B)** PRP added to PVDF/Col NFs; **(C)** PL incorporated into PCL NFs with two different morphologies; **(D)** PRP/PCL core-shell NFs; **(E)** PVA/(Gel/A-PRF) core-shell NFs; **(F)** PCL/CS-PRF core-shell NFs.

Electrospun nanofibers are widely applied due to their unique physical, chemical and biological properties. Possessing high surface area to volume ratios and ECM-mimicking micro/nanoscale architecture, they are ideal for tissue engineering. APC interacts with electrospun fibers diversely; the simplest approach is coating synthetic electrospun fiber surfaces with APC. For instance, PL has been used to functionalize poly (L-lactide-co-epsilon-caprolactone) and poly (epsilon-caprolactone) (PLCL/PCL) nanofibers (Taborska et al., 2023) (Figure 6A). APC forms uniform protein layers on NFs, displaying stability, abundant proteins/GFs and their release. Although most proteins are released from NFs within a week, bioactive molecules like fibronectin, FGF and VEGF persist in the coatings beyond a week due to specific fibrin interactions.

To further enhance APC-electrospinning conjugation, APC can be directly blended into precursor solutions to obtain APC-loaded electrospun fibers. For example, PRP has been added to polyvinylidene difluoride (PVDF)/collagen solutions then electrospun to fabricate PVDF/Col/PRP NFs(Abazari et al., 2020) (Figure 6B). This approach resulted in sustained GF release for up to 14 days without significantly altering fiber diameter or structure, and multiple incorporated GFs enhanced scaffold bioactivity, cell adhesion, proliferation and differentiation compared to pure PVDF/Col NFs.

Nevertheless, previously reported fiber/scaffold units still cannot spatiotemporally incorporate/release important biomolecules. Various biomolecule incorporation strategies have thus been extensively explored, in particular coaxial electrospinning for integrating bioactive molecules into fibers. This technique relies on core-shell production with two solutions simultaneously electrospun coaxially, enabling biomolecule encapsulation within fibers. Loading sensitive biomolecules in the core protects them from direct solvent exposure and microenvironmental fluctuations during *in vitro* and *in vivo* studies. Coaxial electrospinning is therefore an effective means of protecting APC and enabling controlled GF release. For instance, PRP-loaded HA has been injected into the core of PCL shell NFs, achieving effective 30-day APC GF release (Chen et al., 2023) (Figure 6D). Polyvinyl alcohol (PVA)/(Gel/A-PRF) core-shell NFs have also been fabricated via coaxial electrospinning (Tavakoli et al., 2022) (Figure 6E). Compared to other NFs, PVA/(Gel/A-PRF) NFs have finer diameters, higher porosity and surface area. A-PRF crosslinking with the gel and pure PVA core significantly improves shell tensile stress and elastic modulus. The high hydrophilicity and surface area from A-PRF increase L929 cell adhesion/proliferation and angiogenesis, ultimately accelerating full-thickness wound healing. Similarly, PRF-loaded PCL/chitosan (PCL/CS-PRF) core-shell nanofiber scaffolds have been developed using coaxial electrospinning (Rastegar et al., 2021) (Figure 6F). PRF addition to PCL/CS scaffolds decreases PCL/CS-PRF core-shell NF diameter and swelling ratio while increasing porosity, hydrophilicity and degradability. With CS-PRF particles, the PCL/CS-PRF core-shell NF elastic modulus is 1.5 times greater than pure PCL/CS scaffolds. Owing to inter-layer bonding, the PCL/CS-PRF core-shell NF tensile strength exceeds PCL/CS scaffolds. Additionally, the core-shell structure significantly protects PRF, enabling gradual, sustained GF release.

Based on functional gradient concepts, APC-biofunctionalized fibers with distinct morphologies that mimic native layered tissue structures while controllably releasing important biomolecules for tissue regeneration can also be fabricated. Using coaxial electrospinning, biomolecules (PL) have been incorporated into PCL NFs with two different morphologies–anisotropic fibers assembled into yarns (A-Yarn/PL) recapitulating tendon hierarchy and isotropic threads combined with nanohydroxyapatite (nHAp) (I-Thread/PL) to mimic bone organic/inorganic phases (Calejo et al., 2022) (Figure 6C). This enables localized sustained delivery of GFs, cytokines and chemokines. Bioactivity is retained during physiological (neutral pH) and inflammatory (acidic pH) conditions. 3D functionally graded scaffolds comprising editing techniques produce layered structures with composition/morphology gradients, spatiotemporally conveying bioactive cues and eliciting phenotype gradients with cartilage formation and osteochondral features at scaffold interfaces.

### 4.4 3D printing-based APC modification

Bioprinting is an additive manufacturing process capable of fabricating fully functional tissues. 3D-printed scaffolds possess unique advantages including porosity benefiting nutrient/waste transport and customized structures (Akbari and Khademhosseini, 2022). Bioinks, printable biomaterials that can carry cells and biomolecules, play vital roles in bioprinting. However, bioinks themselves remain a major limitation, requiring characteristics to print complex tissues via 3D bioprinting while conferring specific biological cues to support *in vitro* and *in vivo* tissue maturation. Various techniques have been explored to enhance scaffold/bioink bioactivity, e.g., incorporating specific ligands, individual ECM components or engineering material surfaces to promote cell adhesion/vascularization. However, these materials often emphasize enhancing bioactivity for specific developmental stages (cell adhesion, GF-induced vascularization). Spatiotemporal presence of multiple bioactive components is required to support the orchestrated cascade of engineered tissue development (De Santis et al., 2021). Therefore, combining APC with 3D printing is an ideal strategy.

**FIGURE 7:**
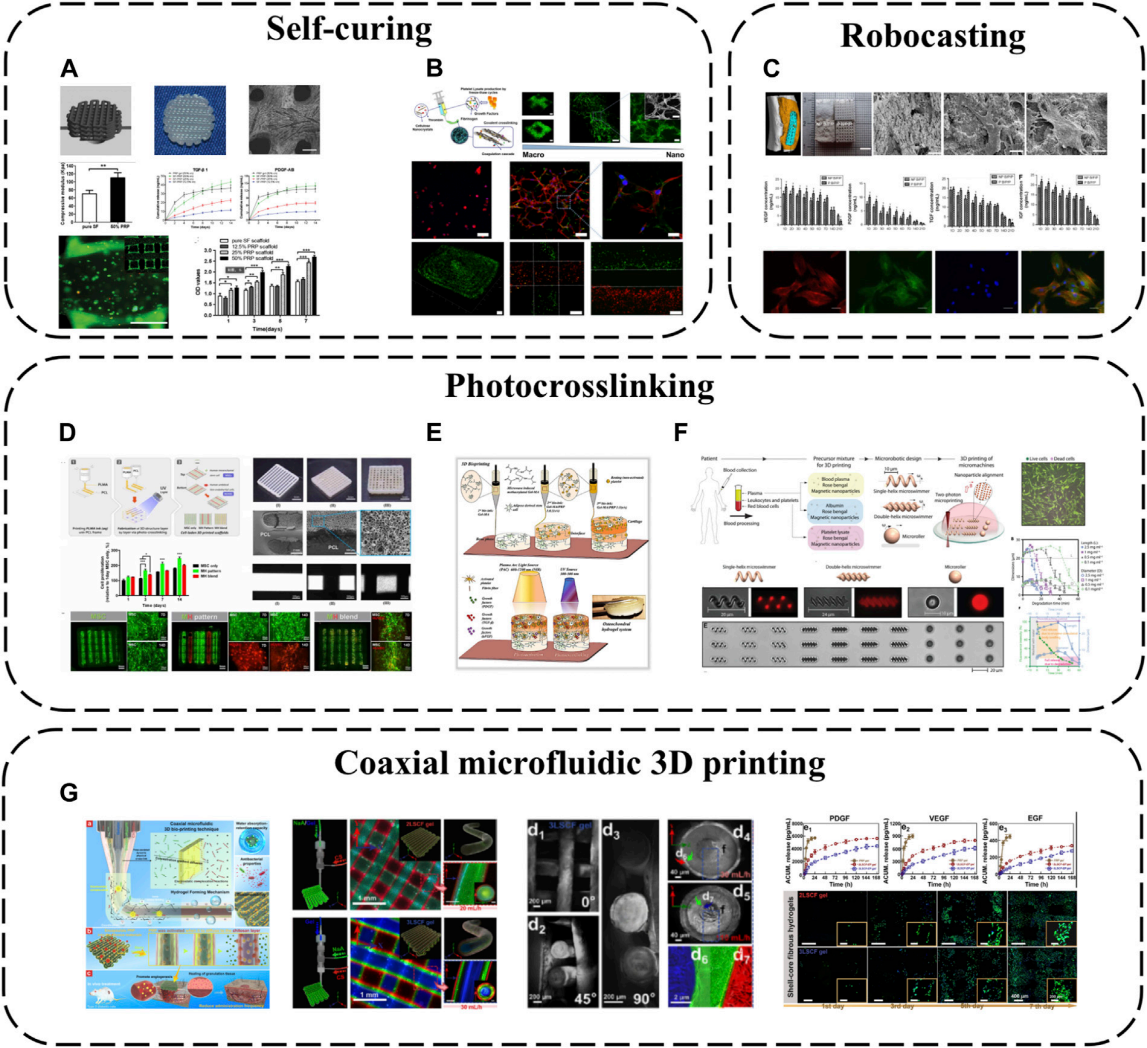
3D printing-based APC modification: **(A)** PRP incorporated into SF bioinks; **(B)** Combination of PL and CNC; **(C)** The combination of BC, PVA and PRF for robocasting; **(D)** PRP has been modified with methacrylate to create photopolymerizable materials (PLMA); **(E)** PRP and GelMA have been combined as bioinks; **(F)** Two-photon polymerization-based 3D printing; **(G)** Coaxial microfluidic 3D printing.

SF is one of the most widely used biopolymers in 3D bioprinting, but its application is limited by insufficient inherent bioactivity. As an autologous GF source, PRP can be incorporated into SF bioinks to improve biofunctionality (Li et al., 2020) (Figure 7A). SF-PRP hydrogel can achieve the controlled release of GF within 14 days. While PRP constitutes half the volume, interestingly, the final SF-PRP hydrogel storage modulus reaches ∼95 kPa, slightly higher than pure SF hydrogels, owing to platelet crosslinking networks in PRP. Beyond SF, CNC can be used to enhance PL hydrogels, forming biofunctional human-based nanocomposite bioinks (HUink) (Mendes et al., 2019) (Figure 7B). In this system, CNC acts not only as a nano-filler trapped in the fibril structure of PL, but also as a cross-linking agent for cross-linking with the amine group of PL protein through reversible Schiff base bonds. The resulting nanocomposite hydrogel has adjustable mechanical and biochemical properties, and there are sulfate groups on its surface, which can effectively bind GF through electrostatic interaction (De France et al., 2017). This strategy makes APC not only as a source of signal biomolecules involved in wound healing, but also as biomaterials with adjustable biophysical and biochemical clues for cell delivery in tissue regeneration strategies. Combined with suspension bioprinting, HUink prints high-resolution, high-integrity 3D freeform structures that mimic native hierarchical tissue fiber assembly from nano to macro scales. HUink supports stem cell bioprinting without additional biochemical/xenogenic media supplementation. Its intrinsic physicochemical/biological cues enable rapid stem cell proliferation and self-ECM production.

As an emerging 3D printing technique, low-temperature robotic casting (robocasting) enables fabrication of geometrically complex ceramic scaffolds at low temperatures, a significant advantage over other additive manufacturing methods that helps preserve APC bioactivity during incorporation. Using this method, nano-biphasic calcium phosphate (BCP), PVA and PRF have been combined to print 3D ceramic scaffolds with ideal pore sizes, porosities and mechanics while achieving 21-day GF release (Song et al., 2018) (Figure 7C). Homogeneously distributed PRF microparticles increase scaffold surface roughness and cell adhesion via anchoring points. PRF introduction markedly improves hydrophilicity and surface roughness, increasing cell seeding efficiency.

Photo-crosslinking is also a common 3D printing approach. GelMA is often employed as a bioink owing to its biomimetic structure and ease of use, photocrosslinking into predefined geometries upon UV exposure. PRP has been modified with methacrylate to create photopolymerizable materials (PLMA) with tunable mechanical properties (Santos et al., 2018) (Figure 7D), circumventing poor standalone APC stability and *in vitro* integrity. This modification generates PL-derived photocrosslinkable hydrogels with adaptable biochemical/mechanical properties. Compared to other PRP/PL materials, PLMA hydrogels possess superior mechanics, *in vitro* stability and support encapsulated cell adhesion/proliferation. Based on these merits, PLMA has potential as a bioink. 3D-printed PLMA scaffolds with suitable porosities/mechanics can replicate customized tissues via simple light-mediated polymerization tuning UV exposure time and bioink concentration. Complex multicellular microenvironments and tissue structures can be mimicked (Min et al., 2021). Additionally, PRP and GelMA have been combined as bioinks for 3D bioprinting and light-activation, developing osteochondral specificity (Irmak and Gumusderelioglu, 2021) (Figure 7E). GelMA possesses similar structure to type I collagen while PRP confers osteochondral bioactivity. This provides a conducive microenvironment guiding stem cell osteochondral differentiation. PRP is dually activated–platelets interact with GelMA structures via integrin receptors, initiating platelet activation. Light stimulation also triggers activation by increasing intracellular calcium release, enabling sustained/controlled GF release. Released calcium reciprocally promotes fibrin network formation, increasing hydrogel crosslinking and stiffness. These light-activatable hydrogels exhibit relatively low degradation, high mechanics and tissue-specific biomimetic structures, promoting *in vitro* osteochondral tissue development.

Since methacrylate/acrylate groups may confer potential toxicity and immunogenicity, APC, rose bengal and magnetic iron oxide nanoparticles can alternatively be combined into precursor mixtures for two-photon polymerization-based 3D printing (direct laser writing), fabricating various geometrically complex medical micromachines like microswimmers and microrollers (Ceylan et al., 2021) (Figure 7F). As a cytocompatible photoinitiator, rose bengal enables direct photocrosslinking of biological macromolecules without requiring additional potentially toxic acrylate/methacrylate groups, while the iron oxide nanoparticles act as magnetic sensors to provide external propulsion. Employing APC as the primary structural material greatly reduces biocompatibility risks. These micromachines display torque-driven controllable motility and shape memory in response to alternating magnetic fields and pH, enabling controlled bioactive molecule delivery/release. They also undergo enzymatic degradation, mitigating long-term inflammation/toxicity risks.

Besides the above printing modalities, coaxial microfluidic 3D printing confers all the general advantages of 3D printing while better preserving APC bioactivity owing to the internal core-shell fiber structures. For instance, CS, SA and gelatin (Gel) have been combined via one-step coaxial microfluidic 3D bioprinting and rapid dual ionic/covalent crosslinking to fabricate multi-layered core-shell fibrous hydrogels (Huang et al., 2023) (Figure 7G). These hydrogels possess excellent hydrophilicity, antimicrobial activity and biocompatibility. Importantly, the multi-layered core-shell structure ensures the PRP core is not prematurely activated during preparation. PRP activation only occurs upon diffusion to the outer CS layer during application, improving GF utilization efficiency. Multi-layered core-shell confinement enables sustained GF release. Compared to clinical PRP gels, these bioactive fibrous hydrogels exhibit controlled GF release, effectively reducing dosage frequency by 33% during wound treatment.

### 4.5 Simplicity is best–APC-based nanomaterial modification strategies

APC provides a rich autologous GF source that can be readily obtained from peripheral blood under the highest safety standards. Tissue regeneration can be effectively achieved by harnessing the body’s own blood-derived resources for personalized regenerative medicine. Conventional tissue engineering therapies like cell therapies have very stringent safety requirements and regulatory hurdles, involving intensive and complex *ex vivo* cell manipulations, time-consuming and expensive procedures that may only be conducted by a small number of highly specialized clinical centers. APC strategies are therefore more easily clinically translatable owing to their simplicity, feasibility, safety and regulatory profiles. They are nearing or already in clinical use, successfully treating patients via unique personalized medicine concepts (Huang et al., 2023). Driven by the principle of “simplicity is best,” an emerging approach is APC-based modification with small doses of nanomaterials to overcome inherent limitations and elicit qualitative changes.

**FIGURE 8:**
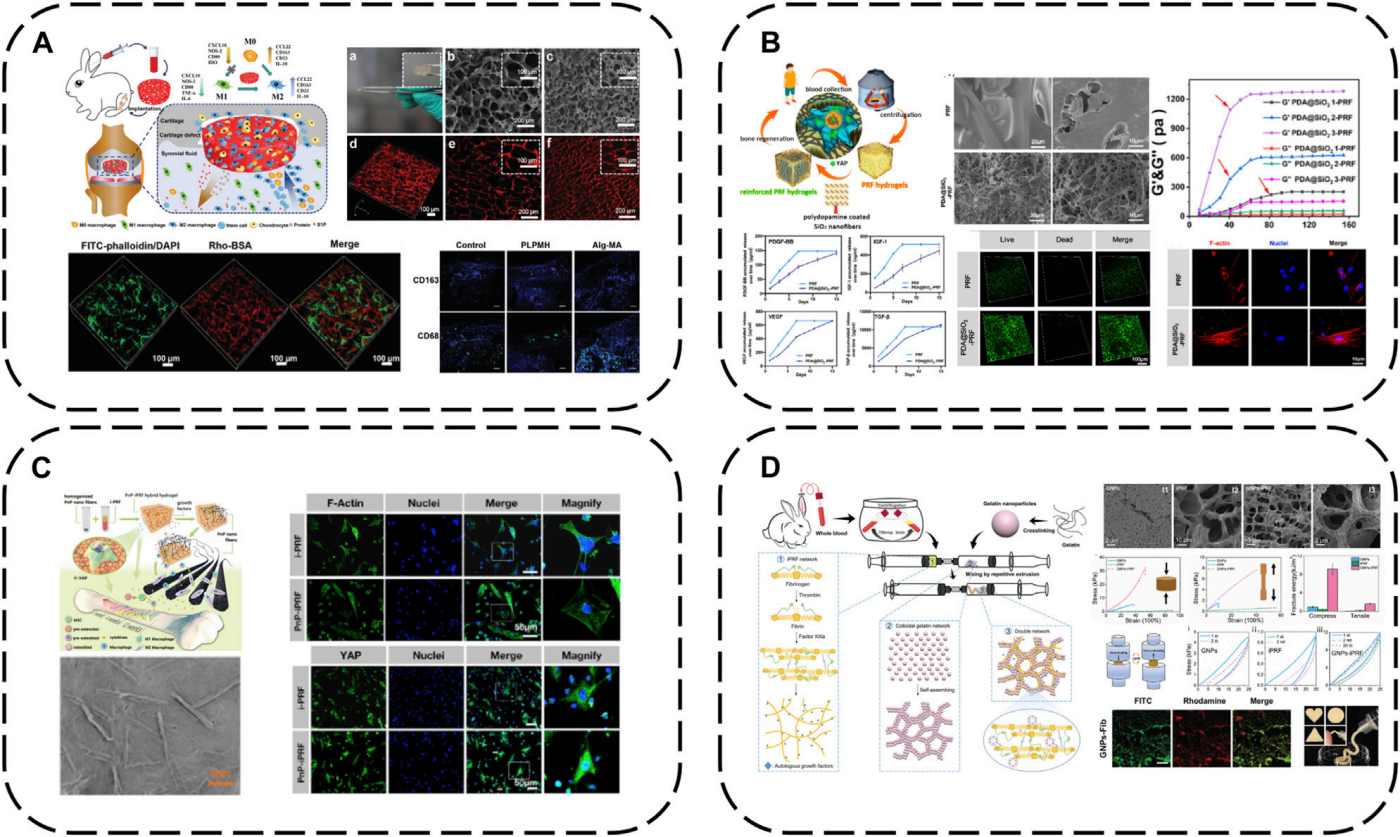
Modification strategy of biomaterials based on APC: **(A)** macroporous PL hydrogels (PLPMHs); **(B)** iPRF modified with PDA@SiO_2_; **(C)** iPRF modified with PnP; **(D)** iPRF modified with GNPs.

APC gels with ECM-mimicking fibrillar structures and abundant biomolecular milieus hold great promise for regenerative medicine. However, poor structural stability severely restricts their use as bioinstructive biomaterials. Therefore, macroporous PL hydrogels (PLPMHs) with ∼100 μm pores and 1.25 MPa Young’s modulus have been developed (Pan et al., 2022) (Figure 8A). Ultrapure alginate crosslinks proteins in PL-rich plasma to carboxylic acids on alginate activated with EDC/NHS. Large pores form via freeze-drying induced water crystallization. These scaffolds contain >95% PL, ensuring biosafety and treatment personalization. Tailored mechanical properties match native cartilage tissue repair/regeneration. Appropriate degradability and large pores enable long-term bioactive molecule release, recruiting endogenous immune and stem cells for activation. This provides localized anti-inflammatory microenvironments for up to 42 days.

The cell mechanical niche is considered a key developmental/growth regulator. Recently, substrate elasticity has been shown to definitively direct stem cell fate - rigid matrices are essential for osteogenesis. Insufficient APC mechanical rigidity therefore accounts for unsatisfactory osteogenic differentiation and bone regeneration efficacy. SiO_2_ possesses uniform nanofibers, high rigidity/toughness and osteogenic capacity via mechanotransduction, with marked *in vitro* and *in vivo* biocompatibility. Inspired by these findings, iPRF has been reinforced with SiO_2_ nanofibers and PDA into meticulously designed autologous protein hydrogels (PDA@SiO_2_-PRF) comprising only 2.5% non-self-components (Ren et al., 2022) (Figure 8B). PDA interlinks iPRF fibrin networks and uniform SiO_2_ nanofibers, tremendously enhancing network mechanics to overcome inadequate rigidity. PDA@SiO_2_ preserves fibrin network structural integrity, impedes rapid PRF degradation and immobilizes GFs, working synergistically to retard burst PRF GF release. Injectable in under a minute, PDA@SiO_2_-PRF is ideal for minimally invasive irregular bone defect filling. Biophysically, it bears loads to maintain bone regeneration space with high rigidity. Notably, the reinforced composite structures possess bone ECM-like functionality, stimulating osteogenic differentiation via the YAP pathway. Biochemically, sustained autologous GF release confers enduring osteogenic capacity.

Similarly, immunomodulatory blood-derived hybrid hydrogels (PnP-iPRF) have been developed by incorporating iPRF into PDA-decorated PCL/hydroxyapatite (HA) composite nanofibers to serve as multi-channel bone microenvironment modulators for enhanced bone regeneration (Li et al., 2022a) (Figure 8C). Beyond tuning the bone immune microenvironment by promoting macrophage M2 polarization to boost osseointegration, PDA also acts as a focal point interfacing the iPRF fibrin network and reinforcing PCL/HA nanofibers to increase rigidity for a conducive biomechanical microenvironment, while the nanoscale HA inherently stimulates osteogenesis. PDA additionally immobilizes iPRF GFs, creating a favorable biochemical niche for sustained osteogenesis. Hence, PnP-iPRF possesses multi-channel capacities to effectively and continuously promote bone regeneration.

Gelatin nanoparticles are also a good choice for eliciting marked APC improvements with small doses. Mixing gelatin nanoparticles with liquid iPRF enables a dual network mechanism–fibrin network formation occurs via fibrinogen-thrombin interactions while electrostatic interactions anchor gelatin nanoparticles onto the networks. Meanwhile, gelatin nanoparticles also spontaneously form non-covalent networks. This mechanism enhances hydrogel mechanics by orders of magnitude compared to standalone APC, conferring a period of injectability for irregular defect-filling and pressure-bearing capacity during bone regeneration, while also prolonging iPRF GF release (Mu et al., 2020) (Figure 8D).

## 5 Prospect

In summary, compared to conventional biomaterials for modern tissue engineering, all APC components are autologous with optimal endogenous ratios and maximal biosafety, enabling personalized medicine. However, standalone APC application has some shortcomings. Combining APC with biomaterials is therefore a viable synergistic strategy, modulating selective spatiotemporal signaling molecule delivery to coordinate tissue regeneration. Considering the complex wound healing/regeneration microenvironment, multifaceted biomolecule delivery will likely be more efficacious than single types.
